# Personal protective equipment preservation strategies in the covid-19 era: A narrative review

**DOI:** 10.1016/j.infpip.2021.100146

**Published:** 2021-05-08

**Authors:** Kiran Grant, James E. Andruchow, John Conly, Daniel Dongjoo Lee, Laurie Mazurik, Paul Atkinson, Eddy Lang

**Affiliations:** a Faculty of Medicine, University of Toronto, Toronto, ON, Canada; b Departments of Emergency Medicine and Community Health Sciences, Cumming School of Medicine, University of Calgary, Calgary, AB, Canada; c Department of Medicine, Cumming School of Medicine, Snyder Institute for Chronic Diseases and O'Brien Institute for Public Health, University of Calgary and Alberta Health Services, Calgary, AB, Canada; d Department of Microbiology, Immunology and Infectious Diseases, Cumming School of Medicine, University of Calgary, Calgary, AB, Canada; e University of Toronto, Faculty of Medicine, Department of Medicine, Division of Emergency Medicine, Toronto, ON, Canada; f Dalhousie University, Faculty of Medicine, Department of Emergency Medicine, Saint John, NB, Canada

**Keywords:** Personal protective equipment, COVID-19, SARS-CoV-2, Reuse, Extended use, Decontamination

## Abstract

**Background:**

The COVID-19 pandemic has led to personal protective equipment (PPE) supply concerns on a global scale. While efforts to increase production are underway in many jurisdictions, demand may yet outstrip supply leading to PPE shortages, particularly in low resource settings. PPE is critically important for the safety of healthcare workers (HCW) and patients and to reduce viral transmission within healthcare facilities. A structured narrative review was completed to identify methods for extending the use of available PPE as well as decontamination and reuse.

**Methods:**

Database searches were conducted in MEDLINE and EMBASE for any available original research or review articles detailing guidelines for the safe extended use of PPE, and/or PPE decontamination and reuse protocols prior to September 28, 2020. Grey literature in addition to key websites from the Centers for Disease Control and Prevention (CDC), World Health Organization (WHO), Infection Prevention Association of Canada (IPAC), and the National Health Service (NHS) was also reviewed.

**Results:**

Extended use guidelines support co-locating patients with confirmed COVID-19 within specific areas of healthcare facilities to enable the use of PPE between multiple patients, and reduce PPE requirements outside these areas. Decontamination strategies for N95 respirators and face shields range from individual HCWs using conventional ovens and microwave steam bags at home, to large-scale centralized decontamination using autoclave machines, ultraviolet germicidal irradiation, hydrogen peroxide vapors, or peracetic acid dry fogging systems. Specific protocols for such strategies have been recommended by the US CDC and WHO and are being implemented by multiple institutions across North America. Further studies are underway testing decontamination strategies that have been reported to be effective at inactivating coronavirus and influenza, and on SARs-CoV-2 specifically.

**Conclusions:**

This narrative review summarizes current extended use guidelines and decontamination protocols specific to COVID-19. Preserving PPE through the implementation of such strategies could help to mitigate shortages in PPE supply, and enable healthcare facilities in low resource settings to continue to operate safely for the remainder of the COVID-19 pandemic.

## Introduction

As communities continue to work to address the Coronavirus Disease 2019 (COVID-19) global pandemic, health systems are at risk of exhausting supplies of critical personal protective equipment (PPE) such as surgical masks, N95 respirators, face shields, goggles, gowns and gloves which are instrumental in controlling the transmission of the virus [1]. PPE is critically important for healthcare workers (HCW) to reduce both their risk of contracting the infection and serving as a potential vector for transmission of the Severe Acute Respiratory Syndrome Coronavirus-2 (SARS-CoV-2) [2]. Preventing infection amongst HCWs is also critically important because, unlike the majority of the population that can practice social distancing, HCWs have many close physical interactions with colleagues and patients on a daily basis posing both an increased infection risk to other HCWs and vulnerable patients [3]. Evidence from Italy suggests that while HCWs are generally younger and healthier members of the population, many have still been infected. Over 300 HCWs have died from COVID-19 in the United States and the global death toll for HCWs is estimated to be over 1,000 [4,5]. Although infections may have been community-acquired, nosocomial acquisition was responsible for some of these infections which may have been preventable with adequate supplies of PPE [1,2]. Current modeling suggests that both many developed and developing countries around the world may face PPE shortages in the coming months [1,6,7]. PPE shortages will likely be more severe in countries with less developed healthcare systems and fewer resources [8]. Consequently, this structured narrative review provides a review of key PPE preservation strategies, including conservation, extended use, decontamination and reuse.

## Methods

Our search strategy was designed to identify any type of extended use, reuse or preservation of PPE with a focus on medical masks, N95, or equivalent, respirators and gowns. A search strategy of two medical bibliographic databases (MEDLINE, EMBASE) was conducted by two authors (KG and DL). A structured narrative review methodology was used because it was best suited to the objective of providing a broad overview of the rapidly evolving literature to aid clinicians and healthcare facilities and making decisions regarding PPE preservation. A systematic review methodology was not used due to the rapidly evolving nature the literature and to facilitate providing a broad overview of strategies used across PPE types, manufacturers, pathogens, and decontamination methods. Searches in Medline (Ovid 1946 to September 23, 2020) and EMBASE (Ovid 1946 to September 23, 2020) were conducted for any available original research or review articles pertaining to the decontamination, disinfection, recycling or reuse of personal protective equipment in any healthcare setting. Articles pertaining to non-medical grade PPE, the decontamination of non-PPE items such as surfaces within hospitals, or medical equipment, simulation studies, and non-English language articles were not included. Literature including guidelines and/or recommendations from the Centers for Disease Control and Prevention (CDC), the World Health Organization (WHO), Infection Prevention Association of Canada (IPAC), Health Canada, the National Health Service (NHS) of the United Kingdom, and the European Union (EU), as well as any academic literature referenced by these organizations, was also reviewed. Studies that met inclusion criteria had the relevant data extracted, and summarized in tabular form and in the body of the text. The quality assessment of included studies was completed using the QUADAS-2 tool which is specifically designed to assess risk of bias in studies [11]. Review articles, guidelines from health authorities, and new articles were included in this review, but were not eligible for quality assessment.

## Results

A total of 33 studies met inclusion criteria and were included in this narrative review. Thirty three studies focused on decontamination of N95 masks 11 studies tested the decontamination strategy on SARS-COV-2 specifically with the remaining studies tested decontamination strategies on other virus' such as SARS-CoV-1 and influenza. All but seven of the studies were published since the start of 2020. Thirteen studies described heat based decontamination strategies, nine described ultra-violet light based decontamination strategies, nine described hydrogen peroxide based decontamination strategies, and two described parecetic acid dry fogging decontamination strategies.

Extended use recommendations are based off of guidelines from international health authorities. There are currently no recommendations regarding the reuse of disposable gowns or gloves. The aforementioned decontamination strategies have not been tested on surgical masks, however surgical masks can likely be reused safely once they have been placed in an open container for 72 hours or more [9,10]. One study specifically tested ultra-violet light in the decontamination of face shields, but otherwise recommendations for decontamination of face shields and other forms of eye protection are based on guidelines from international health authorities.

### Quality assessment

Table 1 summarizes the results of the assessment for each study. 20 of 27 studies had low risk of bias across all four domains of the QUADAS-2, with 4 studies having one of the four domains rated as a high risk of bias, and 3 studies having high risk of bias for both the index test and reference standard domains (Table 1).

**Table 1:** Studies are labeled by reference number. Each of the four domains of the QUADAS-2 tool is listed below, and risk of bias is reported as low, high, or unclear for each of the four categories.

Reference	Patient selection	Index tests	Reference standard	Flow and timing
[10]	Low	Low	Low	Low
[15]	Low	High	Low	Low
[23]	Low	Low	Low	Low
[24]	Low	Low	Low	Low
[25]	Low	High	Low	Low
[26]	Low	Low	Low	Low
[27]	Low	Low	Low	Low
[28]	Low	Low	Low	Low
[31]	Low	Low	Low	Low
[32]	Low	Low	Low	Low
[33]	Low	Low	Low	Low
[34]	Low	High	High	Low
[35]	Low	High	Low	Low
[36]	Low	Low	Low	Low
[38]	High	Low	Low	Low
[39]	Low	Low	Low	Low
[40]	Low	Low	Low	Low
[42]	Low	Low	Low	Low
[44]	Low	Low	Low	Low
[45]	Low	Low	Low	Low
[48]	Low	Low	Low	Low
[50]	Low	Low	Low	Low
[51]	Low	Low	Low	Low
[52]	Low	High	High	Low
[53]	Low	High	High	Low
[56]	Low	Low	Low	Low

### Extended PPE use

Most PPE currently available in health care facilities is designed for single use (i.e., providing a single episode of care to one patient). Under optimal conditions, a gown, surgical mask, face shield or goggles, and gloves would be donned prior to entering the room of a patient on contact and droplet precautions, care would be provided, and all PPE doffed and then either discarded or placed into the laundry hampers (e.g. reusable gowns) as appropriate. Restricting PPE usage to one patient assessment in this fashion reduces the risk of any pathogen from that patient being transmitted to others via the contact/droplet route. This approach is particularly important when individual hospital wards contain patients that are admitted for different medical conditions and the patient with an infection with a transmissible pathogen could easily transmit it to other vulnerable patients around them either directly or indirectly [12]. However, when PPE supplies are strained as we have seen with the COVID-19 pandemic, and several health care systems have completely exhausted PPE supplies leaving both HCWs and patients at risk, the risk-benefit of PPE extended use and reuse requires re-assessment. Moreover, when many patients with the same infection are cohorted in hospital wards, pathogen transmission between patients becomes less of a concern, especially if only patients are cohorted with the same infection. In this scenario, extending the use of PPE past the “one patient at one time” standard is justifiable [9,13].

There are extended use strategies for surgical masks, N95 respirators, face shields, gowns, and gloves (Table 2). The included studies separated emergency departments and inpatient units into zones or designated areas for patients with confirmed/suspected infection and zones with patients unlikely to be infected to mitigate risks of disease transmission to uninfected patients with extended PPE use [14]. Further, the studies reported that the success of these extended use strategies were contingent on sufficient training and logistical support for HCWs. For example, they reported emphasizing strict hand hygiene, facilitating mechanisms to minimize the amount of times HCWs don/doff PPE (such as for drink/meal breaks), and minimizing transit between high-risk and low risk areas to reduce likelihood of infection amongst HCWs.

**Table 2:** PPE extended use and decontamination strategy recommendations by government health organization as of May 25, 2020. CDC = Centers for Disease Control and Prevention, NIOSH = The National Institute for Occupational Safety and Health, WHO = World Health Organization, UVGI = ultraviolet germicidal irradiation, HPV = hydrogen peroxide vapor, HPGP = hydrogen peroxide gas plasma, ED = emergency department, PPE = personal protective equipment, AGMP = aerosol generating medical procedure, HCW= healthcare worker.

Organization	General	Extended use strategies	Decontamination strategies
CDC and NIOSH [9]	• Extended use of PPE permitted only when supply insufficient to enable all HCWs to follow typical protocols • Decontamination of surgical masks/N95s only recommended if the healthcare facility cannot supply each HCW with 5 items of PPE that can be rotated (repeat use only after 5 days of storage)	• Using N95 masks, surgical masks, face shields, gowns, and gloves between patients confirmed to have COVID-19, provided they are not contaminated with bodily fluids or worn during an AGMP • Used PPE can be placed in open containers (paper bag) when not in use • N95 masks should be reserved for AGMPs when supplies do not permit all HCWs access to an N95	• HPV, UVGI, and microwave steam bags are most effective means of decontaminating an N95 mask. • Disinfectant wipes, UVGI, and HPV/HPGP can be used for face shields repeatedly provided no etching in the surface • Surgical mask safe for reuse after storage in an open paper bag for 72–120 hours [9,10] • Soap and water, bleach immersion, and alcohol based cleaning solutions should **NOT** be used to decontaminate PPE [9,15]
WHO [14]	• Enable extended use of all PPE by co-locating all confirmed COVID-19 patients within areas of EDs and inpatient units	• Using N95 masks, surgical masks, face shields, gowns, and gloves between patients confirmed to have COVID-19, provided they are not contaminated with bodily fluids or worn during an AGMP	• HPV, UVGI, ethylene oxide, moist heat (autoclave systems) can be used to decontaminate N95s • Face shields and goggles decontaminated with soap and water followed by detergent (sodium hypochlorite 0.1%) or alcohol wipes
Health Canada [16]	• Non-medical N95s may be used by HCWs at the discretion of the healthcare facility they work in	• N95s and surgical masks may be used beyond their shelf life	• No specific recommendations
NHS [17]	• Surgical masks may be used for source control, if feasible and if the mask can be tolerated by the patient • PPE used in AGMPs should be **single use only**	• Using N95 masks, surgical masks, face shields, gowns, and gloves between patients confirmed to have COVID-19, provided they are not contaminated with bodily fluids or worn during an AGMP • N95 respirators to be worn by HCWs in high risk areas of hospital such as ICU, ED resuscitation rooms	• N95 respirators and surgical masks can be reused provided they have not been soiled, and still fit. No specific strategies recommended. • Face shields and goggles decontaminated with detergent product either combined/sequentially with a decontamination product as agreed by the local infection prevention and control specialists.
EU [18]	• N95 or N99 respirator to be worn by all HCWs in contact with patient **suspected or confirmed** to have COVID-19 • Differing number of sets of PPE allocated to **healthcare teams** based on severity of patient's illness [19]. • If N95/N99 respirators are not available, surgical masks can be used instead	• Using N95 masks, surgical masks, face shields, gowns, and gloves between patients confirmed to have COVID-19, provided they are not contaminated with bodily fluids	• HPV, UVGI, and microwave steam bags can be used to decontaminate N95 respirators [20]. • No recommendations for decontaminating face shields or goggles

Surgical masks have been shown to be safe to use between multiple patients who have been confirmed to have COVID-19 [14]. Guidelines suggest that surgical masks should be discarded if they become wet or soiled and/or damaged in any manner. To safely store a surgical mask, HCWs should be instructed to fold it in half end to end, so the outwards facing side of the mask folds into itself, thus reducing potential contamination of the container into which it is placed. Moreover, it would seem prudent for HCWs to refrain from reusing the surgical mask for at least 72 hours from initial use given that viable virus has been detected on surfaces up to 3-days later based on the available evidence [10].

Similar guidelines exist for the extended use of N95 respirators. However, if the N95 is worn during an aerosol generating medical procedure (AGMP), it needs to be decontaminated prior to use with another patient (11). WHO and Public Health Agency of Canada guidance suggests that N95s be reserved for such AGMPs and are not required for routine patient contact, potentially making any extended use guidelines less applicable. Gowns and gloves can be also be used multiple times between cohorted patients with confirmed COVID-19, though they should not be stored for use another day or shift [9,14]. For all above articles of PPE, these extended use guidelines do not apply if the article becomes wet or visibly soiled with blood and/or bodily fluids or sustains any damage which impairs function (10). While no such folding is possible with a N95 or face shield, care should be taken to not make contact with the outside surface of the mask while removing it and placing it in an open container, such as a brown paper bag [9]. HCWs must also wash their hands prior to donning/doffing the PPE, and/or placing it in a container.

### PPE decontamination and reuse

PPE decontamination and reuse is another important strategy that can be used to preserve supply. Certain articles of used PPE can be decontaminated, whereby any pathogens possibly contaminating the PPE are inactivated prior to reuse (Table 2). Because most PPE currently available in health care settings is designed for single use and there is limited evidence to date demonstrating optimal decontamination and reuse protocols, these protocols should be considered a second-line strategy although new studies suggest this strategy may be quite acceptable. A rate limiting step is that PPE can only be decontaminated a fixed number of times before its integrity degrades to the extent that it compromises fit and function (particularly for N95 respirators) and accordingly strict care and quality assurance measures must be taken to safely implement such strategies. Each of the decontamination methods also requires implementation of protocols within healthcare facilities to ensure staff are trained to safely decontaminate their own PPE, or label it and drop it off at a centralized decontamination site [21].

## Respirators

The best studied PPE decontamination strategies have been for N95 respirators, which can be effectively decontaminated using techniques involving heat [[22], [23], [24], [25]], steam [[26], [27], [28], [29]], UVGI [21,[29], [30], [31], [32], [33]], hydrogen peroxide [29,[34], [35], [36]], or peracetic acid [23,35]. They all attempt to balance inactivating potential pathogens on the mask as possible while minimizing damage to the mask itself (Table 3).

**Table 3:** Comparison of decontamination strategies for N95 masks. UVGI =ultraviolet germicidal irradiation, HPV = hydrogen peroxide vapor, HPGP = hydrogen peroxide gas plasma, PAF = Peracetic acid dry fogging system. ∗Implementing these decontamination systems will require a system for collecting and labeling the PPE such that it can be returned to the HCWs (chain of custody), a mechanism for HCWs to pick up their PPE, and a finally a schedule that ensures that a HCWs article of PPE is decontaminated prior to their next shift.

Decontamination strategy	Process outline	Advantages	Disadvantages	Possible cycles (#)
Dry Heat	• Hot air 70 °C for 30 min • Hair dryer (1400w, 50Hz) from distance of 10–20cm [25] • Can be done in conventional oven or blanket warmer if it can reach target temperature [22,23].	• Can be done at home (oven) or using blanket warmers that are present in most hospitals • Shown to be effective at decontaminating SARS-CoV-2 [24]	• Limited number of studies (4 total) • Damages masks after fewer decontamination cycles.	1–20
Wet Heat/Microwave generated steam	• 90 seconds on high-power, in home microwave (for microwaves with 1100W power), followed by <30 minutes for drying. Steam bags designed for disinfecting infant bottles can be used [26,27] • 3 minutes in 1100W microwave over open glass containers filled with water and covered with mesh [28]	• Can be done at home (microwave) using commercially available products (steam bags) or universally available generic glass containers [28] • Unlikely to cause changes in fit, odor, discomfort, or difficulty donning [29]	• Damages masks after fewer decontamination cycles.	3–20
Autoclave∗	• 121°C for 15 min; total cycle time of 40 min (10 min conditioning/air removal, 15 min exposure, 15 min drying/exhaust), though exact protocol depends on the machine used [23]. Alternative protocol tested 121°C for 30 min with a ∼90 minute total cycle time [37]. 110°C fr 30 min (gravity cycle)	• Utilizes existing autoclave infrastructure present in many hospitals. • Can decontaminate hundreds of masks concurrently	• Damages masks after fewer decontamination cycles.	5-10 [38]
UVGI∗	• Two 254nm UV light sources from two different angles for 5 minutes in dedicated room [30]. Alternative protocols used 1 UV light source (550nm) for 60 minutes [39], 60–70 seconds at 254nm UVGI [33]. • PPE has to be positioned such that there is no shadowing that would prevent full UV light exposure.	• Highly effective, shown to be effective in decontaminating SARS-CoV-2 specifically. • Can decontaminate hundreds to thousands of articles of PPE concurrently.	• Requires specialized equipment and dedicated staff • Total UVC exposure over 950J/cm^2^ may damage N95s after fewer decontamination cycles [40]	30–50
HPV∗ and HPGP∗	• HPV: 1hr cycle consisting of 10 min dehumidification, 3 min conditioning (5 g/min), 30 min decontamination (2.2 g/min) and 20 min aeration. Peak VHP concentration was 750 ppm [23]. • HPV: 5 and 10 cycles of VHP by the V-PRO maX Low Temperature Sterilization System [36] • HPGP: A standard 47-minute cycle using STERRAD® 100NX sterilizer (Advanced Sterilization Products, Irvine, California) [23].	• Highly effective, shown to be effective in decontaminating SARS-CoV-2 specifically. • Can decontaminate thousands of articles of PPE concurrently. • Modular, mobile decontamination unit recently FDA approved.	• Requires specialized equipment and dedicated staff. • Limited number of facilities currently available	30–50
PAF∗	• Need 80–90% humidity (requires approximately 30 ml of dilute liquid peracetic acid for 400ft3 container). Then expose N95 for 1 hr [23].	• Highly effective, shown to be effective in decontaminating SARS-CoV-2 specifically.	•Limited number of studies. • Requires specialized equipment that needs to be frequently cleaned	>10

### Heat and humidity

Pilot studies have experimented with decontaminating N95s using dry heat. The advantage of such a technique is that it could be done in standard blanket warming ovens in hospitals, or at home in a conventional oven, or using handheld hair dryers. While there is no consensus on the specific temperatures and duration needed, studies have demonstrated dry heat of 70–100^0^ C for 30 minutes to have similar level of decontamination as UV light, without compromising mask fit or function [22,39]. With regards to the number of times such a process could be repeated, a pre-publication report from Stanford describes a protocol that successfully inactivated *E.coli* using 75^0^ C heat for 30 minutes for up to 20 total cycles [15]. Another study found that both dry and moist heat at 70^0^ C was effective at disinfecting SARS-CoV-2 and maintained fibre diameter, fit, filtration efficiency, and breathing resistance after 10 cycles [24]. However, a study using hair dryers found that filtration efficiency was reduced after two cycles [25]. Further testing of several heat based decontamination techniques on SARS-CoV-2 is currently underway, including a protocol using dry heat at 75^0^ C for 30 minutes that is part of an international 13 site study in partnership with the WHO [41].

Microwave generated steam bags have also been shown to be effective in inactivating influenza virus and a viral pathogen surrogate, MS2, without compromising mask fit or function, for between 3 and 6 decontamination cycles [26,42]. This approach could be completed at home, as microwave steam bags, which are typically used for decontaminating infant bottles and breast pumps, are commercially available, and most HCWs have access to a microwave. Additionally, a study from Massachusetts, USA, has found that utilizing universally available materials such as generic glass containers and steam can effectively decontaminate N95 respirators and maintain integrity over 20 cycles [28]. Hospital systems in Alberta and Toronto have also been testing the use of autoclave machines, which use a combination of heat, pressure, and steam, to sterilize N95 respirators in large batches [37,43]. One study suggests up to 400 respirators can be sterilized over a 90 minute cycle, and that they remain safe to use after up to 10 decontamination cycles [23]. Other one found N95s to be safe after autoclaving up to 5x in most cases [38]. This approach is particularly promising because many hospitals already have autoclave machines available, and thus could more easily implement this decontamination process.

### UVGI

Heimbuch *et al.* and Lore *et al.* both evaluated UVGI at wavelengths of 254 nm for 6 and 2 different N95 respirator types, respectively [26,44]. Further work by Heimbuch in 2019 showed that 1 J/cm^2^ of UVGI inactivated at least 99.9% of all H1N1, H5N1, H7N9 A/Anhui/1/2013, H7N9 A/Shanghai/1/2013, MERS-CoV, and SARS-CoV that was tested [27]. Likewise, Ozog *et al.* found that 1.5 J/cm^2^ on both sides was effective at decontaminating SARS-CoV-2 [45]. A review by O'Hearn found minimal changes in filter efficiency following application of several different UVGI protocols [46]. Notably, work by Lindsley *et al.* seems to suggest a ceiling on the wavelength of UV light used, as they reported wavelengths above 470nm produced a statistically significant reduction in the strength of the N95 filter [40].

UVGI appears to be an effective way of repeatedly disinfecting N95 respirators. However, instead of a procedure that can be completed by individual HCWs while at home, UVGI decontamination systems would require dedicated funding, space, and technicians, as well as a system for HCWs to drop off and pick-up their specific N95 mask. Schnell *et al.* describe the design of a UVGI system using previous existing components implemented at a hospital in Portland, Oregon [47]. Hamzavi *et al.* has proposed the repurposing narrow-band UVB devices often found in dermatology offices for UVGI [33]. Additionally, a “double hit” process consisting of UVGI followed by heat treatment has been proposed as a conservative method of ensuring maximal decontamination [48].

### Hydrogen peroxide

Hydrogen peroxide can be used as a vapor or gas plasma to decontaminate N95 masks. Hydrogen peroxide vapor (HPV) has been shown to inactivate viruses and highly resistant bacterial spores in the mask and on the mask straps, and is safe to use between 10-50 times per mask depending on the decontamination system used [34,49,50]. Studies by Ibàñez-Cervantes *et al.* and Jatta *et al.* reported that HPGP disinfection on N95 respirators reduced SARS-CoV-2 to undetectable levels after one vapor cycle [36,51]. However, a recent study by Lieu *et al.* tested extended use and HPV decontamination amongst healthcare providers during regular scheduled work hours and found the median number of cycles before respirator failure to be 2, with variation across models, suggesting that failure rate may be faster during real-life work conditions [52]. Hydrogen peroxide gas plasma (HPGP) has been shown to be similarly effective at inactivating pathogens, though less data exist for the maximum of cycles common N95 mask types could tolerate.

Hydrogen peroxide-based systems appear to be quite effective at decontaminating N95s and can be used over many repeated cycles. While these systems would also require significant investment, there is existing infrastructure that can be utilized. Of note, the FDA approved a HPGP system from the Antimicrobial Stewardship Programs to start decontaminating N95s in the US. Additionally, a California-based firm has developed hand-held HPGP devices that have been shown to effectively disinfect N95 respirators with less infrastructure required [53]. A hydrogen-peroxide decontamination process, coupled with strict pick-up and drop-off policies, has been implemented in a large academic hospital in Washington, USA [54] and described for use at the University of New Mexico [55].

### Peracetic acid dry fogging systems

While there is limited literature on the efficacy of peracetic acid dry fogging systems (PAF), they have been shown to effectively inactivate a variety of pathogens, including SARS-CoV-2 specifically, without compromising N95 filter or fit after 10 decontamination cycles [23,35]. However, PAF systems require specialized equipment, and handling of the highly corrosive and flammable liquid peracetic acid.

### Non-recommended decontamination techniques

Notably, there are several means of decontamination that have been recommended against by the CDC [9]. Soap and water, bleach immersion, and alcohol based cleaning solutions have been shown to compromise the N95 filtration efficiency, making any reuse, regardless of the inactivation of any initial pathogens present, unsafe [29].

### Face shields, visors, and goggles

Face shields, visors and goggles are all means of eye protection for HCWs. Generally, face shields are preferred as they can provide broader coverage, and if they cover the full face, can help reduce the risk of surgical masks or N95s becoming soiled or damaged. Provided the face shields are made of a clear plastic material, individual HCWs can clean their own face shield using a wipe and EPA-registered disinfectant [2,9]. If available, face shields could be decontaminated using UV light. A study by Ziegenfuss *et al.* showed that UV light was able to create a 2.4 log reduction in the amount of S. aureus on a face shield using 253.7 nm of light [56].

### PPE collection, storage and redistribution in decontamination protocols

Each of the above decontamination strategies will require clear protocols and training for appropriate PPE collection, decontamination, storage and redistribution. At home decontamination strategies are the least logistically challenging for health systems, but still require HCWs to be trained to safely remove their PPE, store it in a sealed container, transport it home, decontaminate it using their oven or microwave, and then place it in a clean container for transport back to the hospital (see extended use guidelines section for more details). While offering the advantages of possibly greater HCW acceptability and requiring less health system resources and coordination, home-based strategies may be less acceptable to many health systems given the likely higher degree of variability in adherence to recommended protocols and risk for either persistent contamination and damage to PPE, potentially leading to greater infection risk.

In contrast, facility-based decontamination strategies require greater coordination and resources, but can decontaminate hundreds to thousands of articles of PPE concurrently, and remove the burden of protocol adherence from individual HCWs [30]. These protocols generally involve collecting, decontaminating and redistributing individual pieces of PPE to the same HCW that initial used them (a system ensuring chain of custody), encouraging greater end-user acceptability. This is often accomplished by HCWs labeling PPE prior to first use with their name and identification number, date of first use, and a tally mark for number of times reused. HCWs then place used PPE in a labeled container and drop it off at the decontamination center. HCWs later retrieve their personal article of PPE from a centralized pick up location. Hospitals would need to coordinate the schedules of the technicians for the decontamination equipment, porters for transporting PPE through the system, and a reliable means of tracking which articles of PPE belong to what HCW.

## Discussion

While initiatives to redirect all available PPE to healthcare facilities, and rapidly increase PPE manufacturing are underway, maximizing the use of each article of PPE is paramount in the current setting in many jurisdictions around the world. Healthcare facilities should calculate their PPE burn rate to forecast potential shortages (see citation), and then implement PPE preservation strategies as needed [57]. Extended use guidelines suggest that HCWs can safely use surgical masks, gowns, and gloves between multiple patients confirmed to have COVID-19. N95 respirators can be decontaminated effectively using dry heat and steam techniques at home, or at larger scale using autoclave machines. Eye protection, either in the form of face shields and goggles can be cleaned using disinfectant wipes in a manner similar to any hard smooth surfaces. Having individual HCWs disinfect their own PPE places an additional burden on the individual, and requires that they are trained in how to do it properly, however they require less healthcare resources and less coordination than a centralized disinfecting process. For these reasons, it may be better suited to smaller healthcare facilities with fewer resources.

Autoclave, UVGI, HPV, HPGP, and PAF decontamination all require specialized equipment and the creation of a centralized PPE collection, storage and redistribution protocols, however, these processes can be repeated for more decontamination cycles, and can decontaminate larger quantities of PPE at one time. Therefore, these strategies are likely better suited to larger healthcare facilities with the available equipment, staff, funding to decontaminate all HCWs PPE centrally. Depending on local decontamination requirements and available resources, a combination of the centralized and individualized decontamination protocols could be utilized. For all extended use and decontamination strategies, the utmost care should be given to ensuring that all the PPE still fits properly prior to reuse. Effectively mitigating PPE shortages will be critical to preserving health care system integrity by minimizing the number of HCWs and patients infected, particularly in low resource settings.

This narrative review has several limitations. First, while multiple databases were searched, and documents from national and international health organizations were reviewed in detail, no systematic literature search was completed, meaning some relevant studies may have been missed. Second, there were limited studies available describing each of the individual extended use and decontamination strategies outlined, and some recommendations were based on extrapolations of work done on other virus' such as SARS-CoV-1. While the virus' could respond to the decontamination process similarly, more studies on SARs-CoV-2 specifically are needed. Third, while the types of PPE used are quite consistent worldwide, there are many different PPE models and manufacturers, and each product may not respond the same to a given extended use or decontamination strategy. That said, certain manufacturers have started to recommend specific decontamination techniques for their own products, and subsequent studies may help establish if specific extended use or decontamination strategies are not suitable for a given PPE model/manufacturer [58]. Finally, due to the rapidly evolving literature on COVID-19, it is possible that the optimal PPE preservation strategies will change as further testing is completed, and SARS-CoV-2 transmission is better understood.

## Authorship contributions

KG and EL contributed to the conception of this work. KG and DL wrote the initial drafts of the manuscript. All authors were provided a draft of the manuscript for comments and were provided with an opportunity to present revisions. All authors reviewed and approved the final version of the manuscript prior to submission.

## Ethics approval and consent to participate

Not applicable.

## Consent for publication

Not applicable.

## Availability of data and material

Not applicable.

## Conflict of interest statement

The authors declare that they have no competing interests.

## Funding

This narrative review was unfunded.
